# Relation between Level of Serum Transferrin and Postoperative Wound Drainage in Closed Long Bone Fractures

**DOI:** 10.1155/2018/8612828

**Published:** 2018-07-11

**Authors:** Rohan Bhimani, Fardeen Bhimani, Preeti Singh

**Affiliations:** ^1^Department of Orthopaedics, Hinduja Healthcare Surgicals, 11th Road, Khar (West), Mumbai 400052, India; ^2^Department of Orthopaedics, Bharati Hospital, Pune 411043, India; ^3^Department of Orthopaedics, Osmania General Hospital, Hyderabad 500012, India

## Abstract

**Objective:**

To report association between the serum transferrin level and postoperative wound drainage in closed long bone fractures.

**Summary of Background Data:**

There is an old association between the serum transferrin level and wound drainage leading to peri-implant infection. There is no literature available on the ideal treatment for wound drainage. In the majority of the cases, wound drainage usually stops in 3–5 days postoperatively. However, very few cases have been described in the literature about wound drainage following closed long bone fractures.

**Methods:**

A prospective review of the patient's serum transferrin levels and postoperative wound drainage is done.

**Results:**

We reviewed records of 100 patients in whom levels of serum transferrin were checked preoperatively and correlated with postoperative wound discharge. Out of the 100 patients whose serum transferrin levels were checked, 23 patients had low serum transferrin levels and 19 patients had postoperative wound discharge. Out of these 19 patients, 16 patients had low serum transferrin levels. Thus, sensitivity of the test was 84.2% and specificity was 91.3%. In addition, the positive predictive value was 70% and negative predictive value was 96%.

**Conclusion:**

We report that preoperative serum transferrin levels can be used as a good marker to judge postoperative wound drainage in closed long bone fractures.

## 1. Introduction

Peri-implant infection is one of the major causes of morbidity after any fracture fixation. It thus hampers the union of the bone and adds a burden of prolonged immobilization of the affected limb, prolonged hospital stay, neighboring joint stiffness, and so on. Such conditions impose major financial constraints in the outcome of treatment, especially in developing countries.

Most of the wound drainage stops between postoperative days 3 and 5. If it continues after postoperative day 5, it is unlikely to stop spontaneously. There are multiple factors that prolong wound drainage like old age, preexisting malnutrition, increased intraoperative blood loss, obesity, and patients on low-molecular-weight heparin postoperatively. The various treatment options available for the prolonged period of wound discharge are parenteral antibiotics, irrigation, debridement, implant removal, antibiotic-impregnated cemented bead insertion, and so on.

Prevention is better than cure, and thus, if one can anticipate postoperative wound drainage on the basis of certain preoperative markers, then there can be a gross reduction in postoperative morbidity. However, there is very little literature available on preoperative markers to determine whether patients will have postoperative wound discharge. Thus, we decided to use preoperative serum transferrin as a marker to judge whether patients will have postoperative wound discharge in closed long bone fractures.

## 2. Materials and Methods

Between 2015 and 2017, our prospective study included all closed long bone fractures undergoing operative intervention in our institute to find out a factor that could predict whether postoperatively patients are at risk of having wound drainage or not. We excluded all patients with a low serum albumin level. Here, we identified 100 patients of closed long bone fractures undergoing surgical interventions. Using our records, we identified 23 patients who had low serum transferrin levels preoperatively. Of these 100 patients, 19 patients had postoperative wound drainage (that soaked the postoperative dressing) from day 5 onwards. Amongst these 19 patients, 16 had low serum transferrin level (less than 200 mg/dl). All surgical procedures were elective, and no patients displayed evidence of infection in terms of serology or clinical manifestation at the time of surgery. All the procedures were carried out taking all aseptic precautions. The patients were followed up until complete wound healing had occurred, that is, around 1 month.

All surgical procedures were carried out in an operation room with all prerequisites of sterility maintained. We administered prophylactic antibiotic just before the surgery and postoperatively until suture removal. The antibiotic of choice was cephalosporins (cefuroxime and ceftriaxone) and aminoglycosides (amikacin and gentamycin) for 5 days. The postoperative dressing protocol was the same in all patients with the first dressing done on day 2 and the second dressing done on day 5, and intermittent dressings between these two dressings were done if the dressing was soaked. The dressing was done under aseptic precaution using the no-touch technique, povidone-iodine solution, and sterile saline solution. If wound drainage continued for more than 11 days, we posted the patient for debridement. Out of 19 patients, 3 required debridement. Others responded well to regular dressing and antibiotics.

## 3. Results

**Figure 1:**
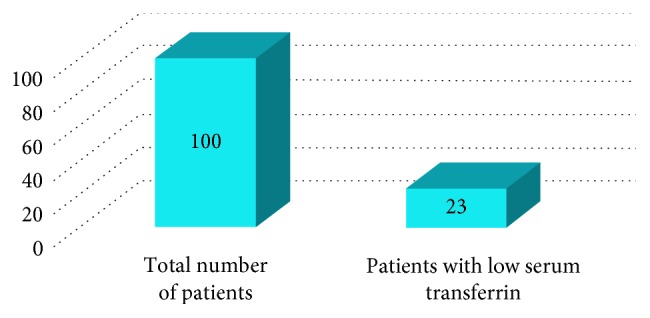
Correlation between the total number of patients and patients having a low serum transferrin level.

**Figure 2:**
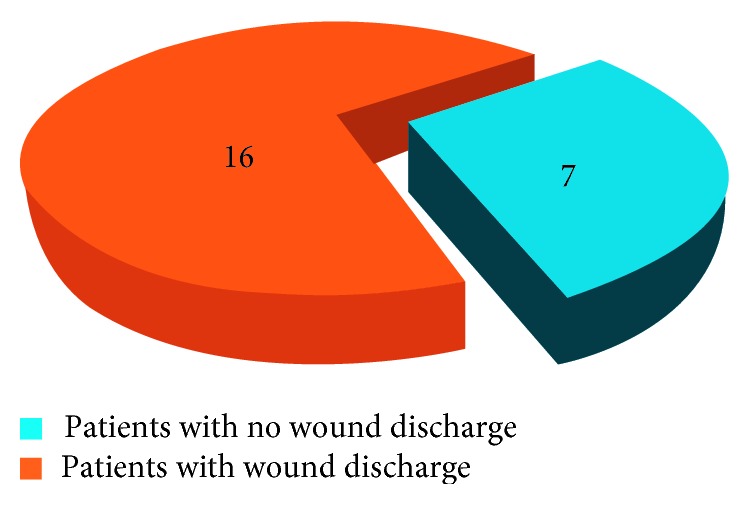
Association between the serum transferrin level and wound drainage.

**Figure 3:**
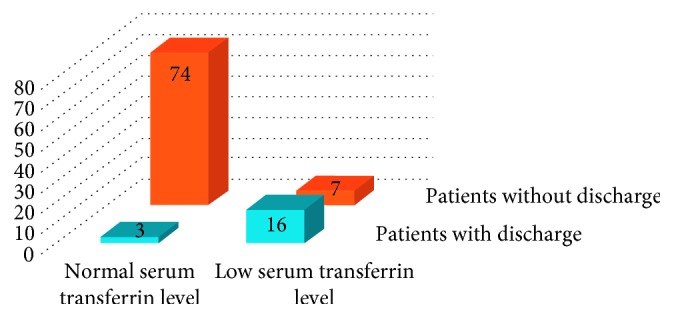
Sensitivity and specificity of serum transferrin.

The total study comprised 100 patients, of whom 23 patients had a low serum transferrin level that is below 200 mg/dl and 19 patients had postoperative wound discharge. Of these 19 patients, 16 patients had a low serum transferrin level (Figures 1 and 2). Thus, sensitivity of the test was 84.2% and specificity was 91.3% (Figure 3). In addition, the positive predictive value was 70% and negative predictive value was 96%.

## 4. Discussion

Transferrins are iron-binding blood plasma glycoproteins that control the level of free iron in biological fluids [1, 2]. The TF gene encodes the human transferrin. Transferrin glycoproteins bind iron very tightly, but reversibly. Transferrin contains two specific high-affinity Fe(III)-binding sites. The affinity of transferrin for Fe(III) is extremely high but decreases progressively with decreasing pH below neutrality. When not bound to iron, it is known as apotransferrin. When a transferrin protein loaded with iron encounters a transferrin receptor on the surface of a cell, it binds to it and, as a consequence, is transported into the cell in a vesicle by receptor-mediated endocytosis. The pH of the vesicle is reduced by hydrogen ion pumps (H^+^-ATPases) to about 5.5, causing transferrin to release its iron ions. The receptor is then transported through the endocytic cycle back to the cell surface, ready for another round of iron uptake. Each transferrin molecule has the ability to carry two iron ions in the ferric form (Fe^3+^). Transferrin is a member of the family which includes blood serotransferrin (or siderophilin, usually simply called transferrin), lactotransferrin (lactoferrin), milk transferrin, egg white ovotransferrin (conalbumin), and membrane-associated melanotransferrin [3, 4]. The liver is the main site of transferrin synthesis, but other tissues and organs, such as the brain, also produce it. It has a half-life of 8 days. The main role of transferrin is to deliver iron from absorption centres in the duodenum and white blood cell macrophages to all tissues. Transferrin helps in transporting iron to the bone marrow for haemoglobin synthesis and to the muscles for storage in the form of myoglobin [5]. Transferrin plays a key role where active cell division occurs [2, 6]. The receptor helps maintain iron homeostasis in the cells by controlling iron concentrations [2, 6, 7]. Transferrin is also associated with the innate immune system. It is found in the mucosa and binds iron, thus creating an environment low in free iron that impedes bacterial survival in a process called iron withholding. The level of transferrin decreases in inflammation [8–10]. During healing, there is active cell division, in which there is a high oxygen demand. Nevertheless, due to decreased haemoglobin, there is decreased oxygen-carrying capacity, and thus, this high oxygen demand remains unfulfilled. This leads to delayed wound healing. In addition, due to its iron withholding property, low transferrin levels lead to decreased bacteriostatic environment, thereby increasing chances of wound infection. Iron also aids in oxygenation of glucose to energy and acts as a cofactor for lysyl oxidase and prolyl oxidase, substances involved in collagen synthesis. Thus, in these cases, collagen synthesis is also hampered.

## 5. Conclusion

Thus, we conclude that serum transferrin is a good marker to preoperatively judge the likelihood of patient developing postoperative wound infection in closed long bone fracture cases.

## Data Availability

All data generated or analyzed during this study are included in this article.
